# Cataphoresis vs. the Direct Application of the Galvanic Current for Obtunding Sensitive Dentine; and How to Conduct a Practice That Excludes Both

**Published:** 1897-08

**Authors:** W. G. A. Bonwill

**Affiliations:** Philadelphia, Pa.


### Cataphoresis vs. The Direct Application of the Calvanic Current for Obtunding Sensitive Dentine; and How to Conduct a Practice that Excludes Both.

BY W. G. A. BONWILL, PHILADELPHIA, PA.

Abstract of paper read before the Dental Section, American Medical Association, Philadelphia, June, 1897.

What have I to say about cataphoresis? Is it a fact that a drug can be dissolved in water or any other media, and be made to traverse the dentinal tubuli, enter the pulp chamber and produce an anesthetic or anelgasic effect by osmosis? Or can it directly paralyze the sensitive dentine upon its surface, and sufficiently deep to enable the operator to cut with impunity painlessly, as is claimed for most obtunders now found in the market? I should not attempt to quibble over osmosis or how or what does produce this supposed effect, but my long experience dating further back than Dr. Richardson, of London, I think entitles me to ask what is the real agent in this wonderful discovery. There are so many means of influencing, not only the human being, but all animals below man, so as to make them believe almost anything you wish even to the complete annulling of pain, that we must be wide awake and very conversant with past history in this line, to enable us to say what agent has produced this so-called cataphoresis, or, as with other pain annullers, what is the true philosophy or *modus operandi* in analgesia.

Osmosis must be established or all such assertions as have been made, fail to convince. Osmosis, according to the best authorities can only take place between two fluids of dissimilar natures or densities, or gravities where a porous membrane intervenes of tissue, or where a porous porcelain cup is used. Then the fluids will in a short time, without any electricity, become of the same strength and gravity. But how can you take the fluid containing cocain and pass it through a membrane that is not porous? Dentine is porous only when the tooth has been extracted and dried, and is void of all organic matter. But so long as it is in the mouth it is full of fluid that is not inter-changeable by osmosis, unless you can produce, either on the pulp chamber and canal, a different density to the fluids in the peridontium.

If equilibrium exist between this medium of dentine on either side of it, then there is a *statu quo* condition and no osmosis. You might as well tell me that a cup can be made of dentine to take the place of the ordinary porous porcelain or burnt-clay cup in a Bunsen Battery, and make of it a battery. Now, I assert that in 1856, I know I did do all this obtunding by the the simple Galvanic current without dam or any other adjunct. Why will you persist in this absolutely useless and unnecessary procedure in loss of time, demoralizing your patients, fooling away your own senses, trying to believe you are doing something that was never done before and is pre-eminently superior to all that was ever done in the past history of dentistry?

It is all well enough to try to alleviate pain in any operation, but when all of this can be done without, and the patient is enabled to see that dentistry is not the inhuman thing dentists would have them believe, and have them feel and know that they can be taught to bear all pain consequent upon any operation on the teeth, in excavating or removing pulp, why should you not adopt means long ago in your grasp for the asking and taking. From the many experiments of others in electrical therapeutics as far back as 1860, osmosis was proven to take place by a current of electricity through a porous membrane or diaphram, but never through bone either in living subject or dead; and even when the osmosis was affected through a porous animal structure, or membrane, or porous cup, it was only done after many hours action. Teeth were extracted in this city, by electricity, as early as 1859, but it was only by the shock produced at the instant the forceps was applied, producing a diversion of the will force by causing a sudden and violent inhalation into the lungs, and while the lungs remainded inflated, the effect was good, for the senses were, for the instant, submerged or subjugated. From my use of the battery and catching on to its working upon myself and patients, I found that the continuous current, or, when interrupted several thousand times a minute, would annul pain when directly applied to the excavating and the negative pole on the face or in the hand and no dam or other agent. All of this work, however, was preceded by experiments directly upon myself in the use of chloroform. These experiments led to the discovery that chloroform could be taken to that extent that I could excavate my own carious cavities without pain and yet be sensible of the sense of touch and ability to perform the operation upon myself. Chloroform, while it would do what I wanted, would make my patients too sick, and I had to abandon its use. It was then that the battery for extractions alone was first brought out in Philadelphia, and I have told you the philosophy of shock in its action on respiration. I soon had it so exemplified to the satisfaction of the patient, that, while electricity would annul pain in sensitive dentine and living pulps (if not inflamed), could be removed, yet from the too strong application of the current it gave the patient such a severe shock while excavating that a violent inspiration was the result which led me to exclaim: "Nature's Anesthetic," and I then saw that it was diversion of the will-power, for when the lungs were being inflated so violently, the will could not take cognizance of actual pain. It was for the instant complete. Let any one hurt a finger and how soon it is put in the mouth and a violent inhalation taken several times until pain is relieved. The infant is crying violently while in pain, from an accident, is relieved and falls to sleep from the constant sobbing and increased inspiration. All temporary teeth I extract by this method—one sudden inhalation or diversion of the will and not a tear or complaint. Two or three teeth can be extracted while the breath is held in the lungs. You now know why I abandoned electricity for obtunding sensitive dentine and extracting, for this revelation of how nature relieves. I am led to assert here today that all this cataphoresis in dentine is only the work of the current pure and simple, and while I can annul pain in a few minutes by the current, I will not fool with it, as, by my present mode of practice, it is no longer worthy of my notice, and could you follow me day by day at the chair, you would adopt the means which would not rob you or your patient of that which neither can ever replace or have paid for remuneratively. I hold it is all nonsense for you to practice deluding yourselves and robbing the public of valuable time, which, unless you get paid for every minute lost in the application of electricity by cataphoresis, you are a loser also.

